# Research progress on antisepsis effect of apigenin and its mechanism of action

**DOI:** 10.1016/j.heliyon.2023.e22290

**Published:** 2023-11-15

**Authors:** Lin Zhu, Hairong Zhang, Xiaoyu Zhang, Lei Xia, JiaJia Zhang

**Affiliations:** a School of Chinese Medicine, Shandong University of Traditional Chinese Medicine, Jinan 250355, PR China; b Shandong Provincial Third Hospital, Shandong University, Jinan 250031, PR China; c Department of Pathology, Shandong University of Traditional Chinese Medicine, Jinan 250355, PR China

**Keywords:** Sepsis, Apigenin, Inflammation, Herb, Mechanism

## Abstract

Sepsis is an abnormal immune response to infections and can trigger MODS. Despite the availability of advanced clinical techniques and monitoring methods, the mortality rate of the disease is still high, posing a heavy burden to patients and the whole society. Hence, the research on novel drugs and targets is particularly important. As a natural phyto-flavonoid, apigenin boasts anti-inflammatory, antioxidant, anti-cancer, anti-viral, and anti-bacterial effects. Besides, *in-vitro* experiments and animal models have also revealed the crucial role of apigenin in the treatment of infectious diseases and sepsis. In this context, this paper reviews the pharmacological activity and underlying mechanisms of action of apigenin in sepsis treatment and organ protection, as well as the potential apigenin-based therapeutic strategies against sepsis. Therefore, this review will shed new light on the scientific research and clinical treatment of sepsis.

## 1 Introduction

Sepsis, a critical disease associated with high morbidity and mortality as well as hefty treatment costs, has been defined as a life-threatening organ dysfunction triggered by the dysregulated host response to infections. Despite the availability of advanced clinical treatment techniques and monitoring measures, the mortality of sepsis is still high, imposing a heavy burden on patients, their families, and the whole society. It was reported that approximately 50 million people suffered from sepsis in 2017 and 11 million of them died, which accounted for about 19.7 % of the global deaths [1]. The pathogenesis of sepsis is multifaceted, It is involved with systemic inflammatory response syndrome, ischemia-reperfusion injury, oxidation and antioxidant imbalance, immune dysfunction, mitochondrial dysfunction, apoptosis and autophagy disorders. In recent years, an increasing number of herbal monomers have been adopted in septic treatment clinically. Natural plant-originated ingredients are vital in septic treatment, as they are cheap and simple to extract characterized by multiple pathways, multiple targets, and bidirectional modulation.

Apigenin is a natural phytoestrogenic flavonoid present in a variety of vegetables and fruits in temperate and tropical zones. As apigenin is particularly abundant in celery, it is extracted from celery seeds as an alkaline ingredient. With diverse biological activities, apigenin is endowed with antioxidant, anti-inflammatory, anti-bacterial, anti-viral, anti-fungal, and anti-cancer effects, thereby demonstrating the value of extensive clinical applications [[2], [3], [4], [5], [6], [7], [8], [9], [10], [11]]. Recent findings have revealed the therapeutic and preventive effects of apigenin against infectious diseases. In this context, this paper intends to explore the role of apigenin in the treatment of sepsis and the underlying mechanisms.

## 2 Survey methodology

By using the advanced search method in Pubmed, CNKI and CSPD database, we entered the subject terms "sepsis" or "LPS" or "CLP" and "The search period was from January 1, 2005 to January 1, 2023, and a total of 79 papers on experimental and clinical studies of apigenin in the treatment of sepsis were retrieved. A total of 66 papers were included to summarise the progress of research on the mechanism and efficacy of apigenin in the treatment of sepsis.Inclusion and exclusion criteria: Inclusion meets the diagnostic criteria for sepsis (clinical patients meet the new criteria for the diagnosis of sepsis from the 2001 International Conference on the Definition of Sepsis, animal studies meet the criteria for cecum ligation and lipopolysaccharide Lps intraperitoneal injection moulding). Interventions: apigenin and apigenin analogues. Studies with incomplete information and duplicate publications were excluded.This article reviews recent research advances in the treatment of apigenin in sepsis and its role *in vivo* and *in vitro* experiment and its mechanisms. This article consolidates the experimental articles related to apigenin. In recent years, recent studies have found that apigenin has both therapeutic and preventive effects in the sepsis. This is an area that should be of interest to us.

## 3 Underlying mechanisms of sepsis in the treatment of sepsis

### 3.1 Anti-inflammation

Early-stage sepsis primarily features the activation of host immune responses and massive release of inflammatory mediators, but excessive inflammatory responses can aggravate the damage and even induce MODS [12]. Following inflammatory priming, an anti-inflammatory response may be initiated in the body as a compensatory mechanism to avoid the damage to the body caused by excessive inflammatory responses. However, this may lead to waning immunity and a higher mortality as a result. When CARS and SIRS are co-existing and mutually reinforcing, inflammatory responses and more severe immune disorders can be triggered, presenting the body with more severe damage. A previous study confirmed the clinical value of inflammatory factors and their changes in the prediction of the prognosis of septic patients [13]. While apigenin was found to have strong anti-inflammatory activity in both *in vitro* and *in vivo* models [[14], [15], [16], [17]]^,^ and the underlying mechanism was associated with the inhibited expression of COX-2 and cellular adhesion molecules as well as the adhesion between monocytes and human umbilical vein endothelial cells [18]. Investigation of oxidant/antioxidant and anti‐inflammatory effects of apigenin on apoptosis in sepsis‐induced rat lung [19]^.^ In a rat model with CLP-induced sepsis, apigenin pretreatments could evidently reduce the number of inflammatory cells, induce the generation of anti-inflammatory factors, and suppress the production of proinflammatory cytokines [20]. Besides, apigenin could also visibly repress the production of pro-inflammatory cytokines such as IL-6 and TNF-a among the LPS-induced septic rat model [21]. An *in-vitro* experiment revealed that apigenin decreased the expression and protein secretion of TNF-a and IL-10 in RAW-264.7 macrophages [22]. Marrassini et al. also reported that apigenin-7-glucuronide, the derivative of apigenin, could exert its anti-inflammatory effect by diminishing the release of TNF-α and nitrite in the LPS-activated macrophage cell line RAW264.7 [23].

**Fig. 1:**
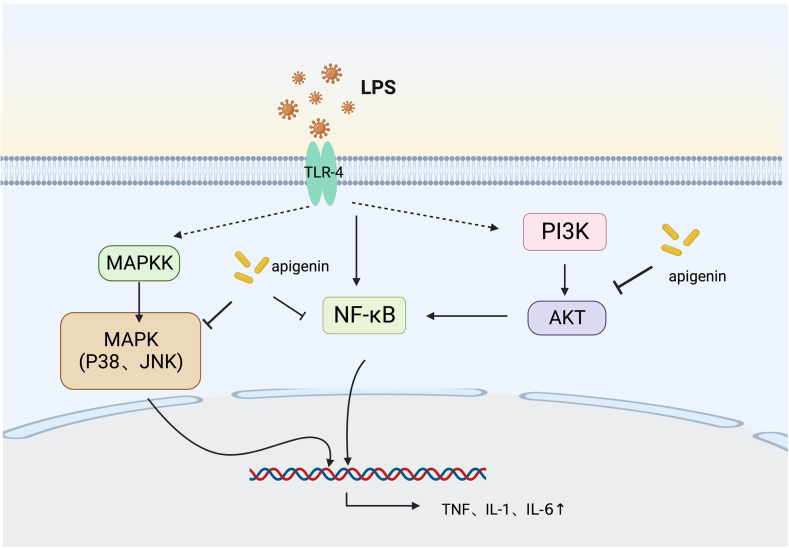
Anti-inflammatory mechanism of apigenin in sepsis. Apigenin reduces the release of inflammatory mediators TNF, IL-1, and IL-6 through MAPK, NF-kB, and PI3K signaling pathways, thereby alleviating sepsis damage.

The NF-κB pathway is considered as a conventional pro-inflammatory signaling pathway. NF-κB is involved in the expression of pro-inflammatory cytokines, including cytokines, chemokines, and adhesion molecules. With the LPS-stimulated monocytes, Nicholas et al. discovered that [24] apigenin obviously suppressed the generation of pro-inflammatory mediators (IL-1, IL-8, and TNF), which was associated with the inhibited transcriptional activity of NF-κB. In contrast, apigenin did not influence LPS-stimulated IKK degradation or the NF-κB-DNA binding capacity, but played its role primarily through the suppression of p65 phosphorylation. In the meantime, a further investigation on NF-κB downstream signaling pathways showed that apigenin, while repressing the activation of NF-κB, could elevate the concentrations of GSK3β, Nrf2, and HO-1 [25]. The LPS-stimulated keratinocytes could also induce the activation of NF-κB through TLR-4 to play its pro-inflammatory and immune role. The activation of NF-κB might be correlated with the PI3K/Akt signaling pathway, while JNK and p38 were also found to be implicated in the activation of NF-κB in various tissues [26,27]. Additionally, Kim et al. demonstrated that [28] apigenin might repress the LPS-induced production of cellular inflammatory mediators by diminishing the activation of TLR-4-dependent Akt, mTOR, and NF-κB pathways as well as JNK and p38-MAPK(Fig. 1).

### 3.2 Antioxidation

**Fig. 2:**
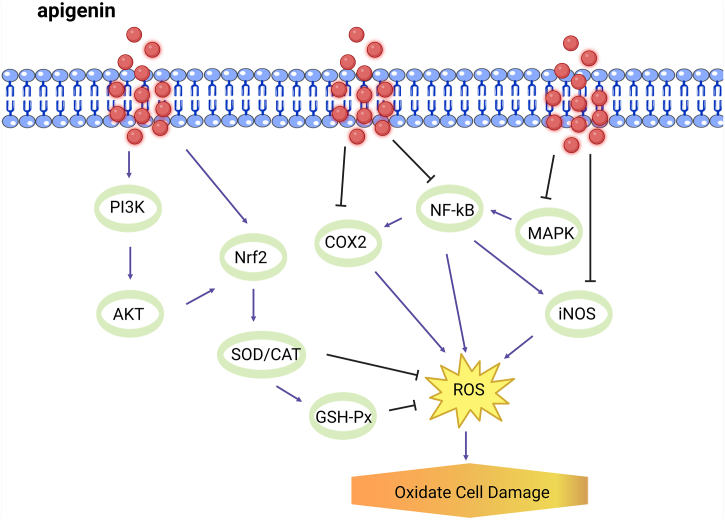
Antioxidant mechanism of apigenin in sepsis. Apigenin regulates ROS production by promoting PI3K and Nrf2 to inhibit MAPK, NF-kB, iNOS, and COX2, and attenuates sepsis injury.

Oxidative stress refers to the imbalance in oxidative and antioxidant homeostasis due to the inability of the body to promptly eliminate the large amount of ROS generated under diverse internal and external factors. Oxidative stress participates in a variety of physiological and biochemical reactions while impairing cellular and subcellular structures. Under sepsis, massive ROS generation induced by the activation of numerous neutrophils is primarily responsible for organ tissue damage [29]^.^ Apigenin and its derivatives, possessing excellent antioxidative effects [30], function in the treatment of multiple diseases by inhibiting oxidative stress. Under the AGE-induced oxidative stress and inflammation in human umbilical vein endothelial cells, Zhou et al. found that [31] apigenin could directly bind to methylglyoxal to repress the production of AGEs, and that apigenin and di-apigenin adduct could evidently inhibit ROS generation and down-regulate the expression of proinflammatory cytokines and adhesion molecules. Their further investigation demonstrated that apigenin exerted its protective effects potentially via the inhibition of the ERK/NF-κB signaling pathway and then the activation of Nrf-2 pathways. Finally, the anti-oxidant stress capacity was enhanced. The antioxidant mechanisms of apigenin also include oxidase repression, regulation of redox signaling pathways (MAPK and P13/Akt), and enhancement of enzymatic and non-enzymatic antioxidants, metal chelation and free radical scavenging [32]. With the help of cellular experiments, Zhang et al. revealed that [33] apigenin increased the expression of SOD, CAT, and GSH-Px, but inhibited the formation of MDA. This might be associated with the enhanced activation of Nrf2 (a significant regulator of oxidative stress) and the lifted expression and nuclear localization of its downstream target genes, because the anti-oxidative stress capacity of apigenin was markedly weakened under Nrf2 suppression. Oxidative stress is one of the important contributors to organ damage under sepsis [34], while LPS exerts the cytotoxicity by increasing ROS production in endothelial cells [35]. Through the mouse model with LPS-triggered myocardial injuries, Fang Li et al. reported that [36] apigenin substantially lowered the levels of oxidative stress products (protein carbonyl and nitrotyrosine) and inflammatory factors (TNF-α, IL-1β, MIP-1α, and MIP-2) in mice, which might be involved with NF-κB suppression. Moreover, with LPD-induced endothelial cellular damage, Durate et al. discovered that [37] apigenin visibly decreased LPS-induced ROS production, which was potentially correlated with the protection of mitochondrial function and restoration of mitochondrial complex I function (Fig. 2).

### 3.3 Macrophages

Macrophages, as one of the most important cell types in the innate immune system, participate in both innate and adaptive immunity with the capacities of polarization, autophagy, and inflammatory response regulation. In recent years, strong evidence has been provided indicating that biological abnormalities such as macrophage polarization and autophagy result in body decompensation and exacerbate inflammatory responses, presenting wide implications on the body during septic development [38].

It was pointed out that apigenin triggered apoptosis among resting macrophages, which was possibly implicated with the increased intracellular ROS, regulation of the MAPK pathway, and consequent repression of Bcl-2 expression [39]. Besides, apigenin may also promote the apoptosis of OxLDL-loaded MPMs through PAI-2 inhibition, while PAI-2 exhibits an anti-apoptotic effect in OxLDL-loaded macrophages [40]. In activated macrophages, apigenin can also exert effects by interfering in cell death or polarity conversion.

**Fig. 3:**
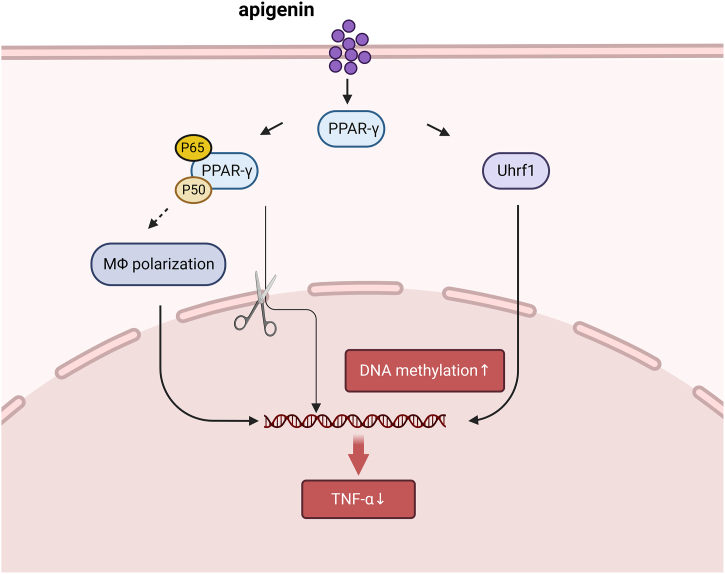
Mechanism of apigenin in the polarization of macrophages in sepsis. Apigenin reduces septic macrophage necrosis in sepsis by modulating the PPARγ pathway, regulating Uhrf1 transcription and blocking p65 translocation.

Alveolar macrophages account for the majority of leukocytes in the airways (approximately 95 %) and are the principal immune cells responsible for lung homeostasis in alveoli [41]. By inducing alveolar macrophage apoptosis with mycoplasma, Mei et al. revealed that [42] apigenin could lower TNF-α levels and protect the macrophages. During this process, apigenin might modulate Uhrf1 transcription through PPARγ activation, thereby inhibiting the expression of TNF-α via Uhrf1-dependent DNA methylation. In addition, PPARγ also serves as a major regulator in macrophage polarization. It was reported by Feng et al. that [34] apigenin bound to the LBD and hinge domain of PPARγ to activate PPARγ, and subsequently the activated PPARγ formed a complex with p65. In this way, the generation of inflammatory factors was diminished, and meanwhile the functional polarization of macrophages was altered by changing the intracellular localization of p65/PPARγ [43]. Apigenin significantly inhibits LPS-induced pro-inflammatory cytokine production by modulating multiple intracellular signalling pathways in macrophages. Apigenin is able to inhibit caspase-1 expression and reduce mRNA stability through NLRP1 inflammatory vesicles and ERK6/1 activation, and inhibits NF-κB activation, thus exerting an anti-inflammatory effect [44] (Fig. 3).

### 3.4 Endothelial cells

One of the hallmarks of sepsis is endothelial dysfunction [45].Severe infections directly impair the vascular endothelium, and induce tissue edema, thrombosis, I/R injury, and so on [46]. Apelin is an endogenous ligand for the G protein-coupled receptor APJ and a variety of physiological processes. It was proved to actively regulate angiogenesis both *in vitro* and *in vivo*, and was also found to stimulate endothelial cell proliferation, migration, and tube formation *in vitro* [47,48]. Yamagata et al. indicated that [49] the endothelial cells exhibited obviously lower levels of apelin after hypoxic reoxygenation, but the declining trend could be reversed by apigenin.

**Fig. 4:**
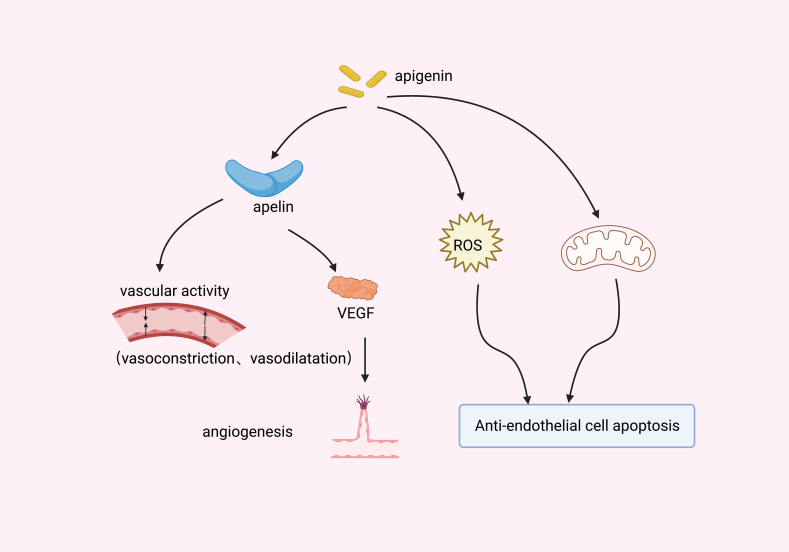
Mechanism of apigenin in endothelial cells of sepsis. Apigenin reduces endothelial cell apoptosis by regulating apelin, ROS and mitochondrial function in sepsis.

It was reported that apigenin suppressed the transcriptional activation of VEGFs in a dose-dependent manner [50]. Furthermore, Durate et al. also pointed out that [37] apigenin, by protecting mitochondrial functions, reduced ROS generation, restricted casp-3 activation, and alleviated LPS-stimulated endothelial cell apoptosis (Fig. 4).

### 3.5 Autophagy

Autophagy is an essential process in eukaryotes for the turnover of intracellular substances. During the early stages of sepsis, increased number of autophagosomes and enhanced expression of autophagy-related proteins can be observed in multi-organ cells. In the CLP model, the expression of the autophagy marker LC3 was markedly up-regulated in multiple organs 6 h postoperatively. While DAMPs and PAMPs could activate autophagy through the activation of NF-kB, MAPK or mTOR pathways via TLRs during pathogenic infections [51].

**Fig. 5:**
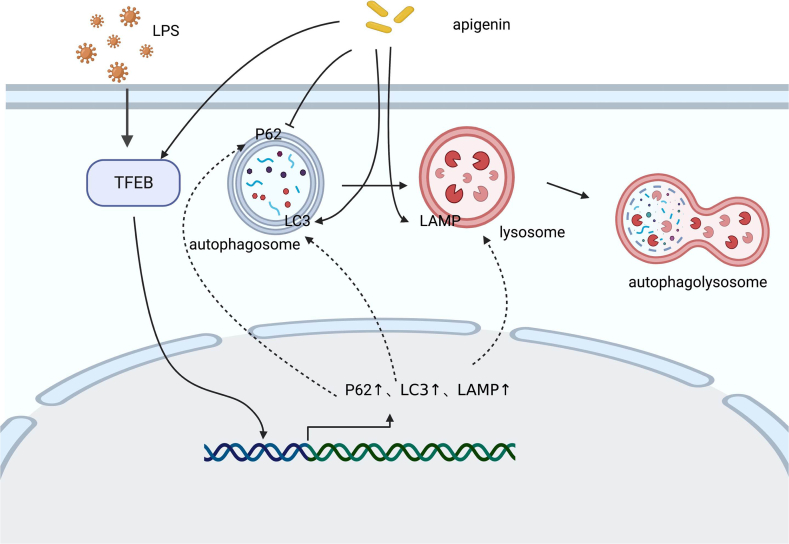
Mechanism of apigenin in sepsis autophagy. Apigenin affects autophagy by modulating the autophagy regulator TFEB, lysosomes and autophagosomes，reducing septic injury.

The modulation of apigenin on autophagy has already been reported. For example, previous research demonstrated that [52]apigenin removes damaged mitochondria through mitophagy and attenuates Hepatic Pyroptosis via the mitochondrial autophagy-ROS-CTSB-NLRP3 pathway and apigenin facilitated the apoptosis and autophagy of liver cancer cells [53]; when the autophagy process was repressed, the tumor cell death-promoting role of apigenin was significantly reinforced. RRRuela-de-Sousa et al. investigated the inhibitory effect of apigenin on different tumor cells, and revealed that [54] apigenin could induce the apoptosis of certain tumor cells, but the pro-apoptotic effect was unobvious among some tumors. This might be attributed to the protective effect on tumor cells brought by the induced autophagy. The pro-autophagic role of apigenin was also reported in a study regarding cisplatin-resistant colon cancer cells [55]. Hence, for cancer patients, the addition of apigenin in food should be determined carefully, and the combination of apigenin and autophagy inhibitors should be considered during treatment [53,54]. It was shown that in the treatment of sepsis, the levels of LAMP1, ATG5, and p62 all went up in the endotoxin-induced myocardial injury model, while apigenin could further elevate LAMP1 and ATG5 levels but lower the level of p62 [36]. The mechanism was possibly correlated with the autophagy regulator TFEB. To be more specific, LPS facilitated the expression of TFEB, while apigenin further increased its expression, and the combination of apigenin and LPS lifted the nuclear localization of TFEB (Fig. 5).

### 3.6 Apoptosis and necrosis

Apigenin can also play the protective role in the body through multiple other mechanisms, such as apoptosis and necrosis alleviation. As previously described, apigenin attenuates I/R-induced cardiomyocyte injury, which may be associated with the inhibition of apoptosis and pyroptosis [56]. Both the *in-vivo* and *in-vitro* experiments of Wang et al. revealed [57] the evident protective role of apigenin pretreatments in renal I/R-related injuries, and they also proved that apigenin diminished the mitochondria-dependent apoptosis via the activation of PI3K/Akt signaling pathways. The research of Zhou et al. on H9C2 myocardial I/R cells also yielded similar results [58]. With the middle cerebral arterial I/R model, Pang et al. demonstrated that [59] apigenin might relieve the apoptosis and autophagy of HBMVECs (endothelial cells) through the Caveolin-1/VEGF pathway to boost cell proliferation, thus improving the nerve function and protecting the brain. Patients with early-stage sepsis generally suffer from circulatory system dysfunction. But during treatment, they are usually confronted with I/R injuries with the functional improvement of the circulatory system.

Apigenin not only interferes with I/R injury-induced apoptosis, but also alleviates tissue damage directly triggered by LPS. Cardenas et al. noted that [60] apigenin diminished the LPS-induced apoptosis in lungs, inflammatory cell infiltration, and chemokine accumulation, thereby reconstructing the normal lung architecture. In the meantime, apigenin also presented with direct attenuating effect on LPD-stimulated endothelial cell apoptosis [26,61]. Li et al. discovered that [62] apigenin C-glycoside could also repress LPS-induced apoptosis of lung epithelial cells under ALI via mitochondrial pathways. The mechanism was potentially involved with the effective obstruction of p38, ERK1/2 and JNK phosphorylation.

**Fig. 6:**
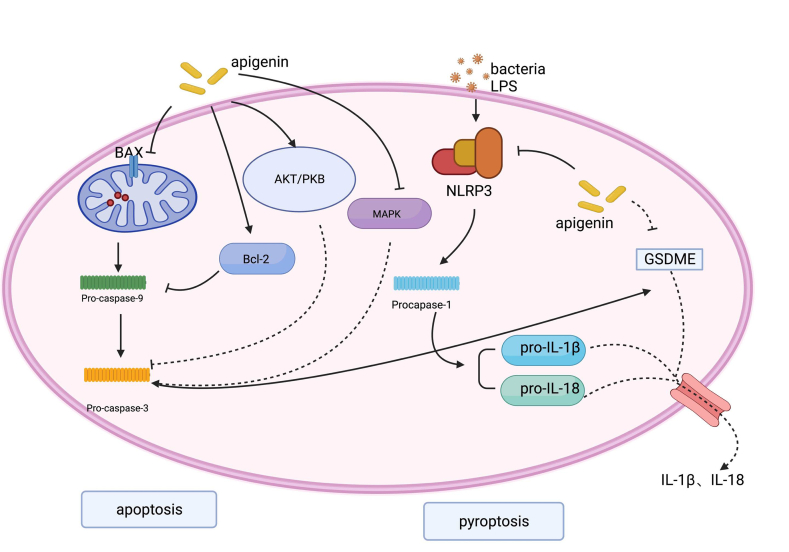
Mechanism of apigenin in apoptotic and pyroptosis in sepsis. Apigenin attenuates sepsis injury by affecting cellular pyroptosis through regulation of the pyroptosis-associated proteins caspase1, NLRP3, and GSDME-N, and by regulating apoptosis through PI3K/Akt/MAPK/BAX/Bcl-2.

Moreover, apigenin was also found to trigger the apoptosis of murine macrophages ANA-1, which might be associated with the MAPK pathway. Although apigenin had no impact on the expression of p38, ERK, and JAK, it could lower the levels of *p*-ERK and *p*-JNK while elevating p-p38 levels [29]. Taken together, apigenin can not only protect the organs of septic patients via the intervention in apoptosis, but also play a modulating role in immune system functions (Fig. 6).

## 4 Protective role of apigenin on organs

### 4.1 Apigenin in sepsis-related cardiac injuries

Septic cardiomyopathy, one of the most severe complications of sepsis, directly impacts the prognosis of septic patients. It was previously reported that the mortality among septic patients with cardiac dysfunction reached 70 %, while the mortality among those without myocardial dysfunction was only 20 % [63]. Therefore, effective cardioprotection is of great significance for elevating the survival rate in septic patients. Apigenin is known to possess therapeutic and preventive effects on various cardiovascular diseases [[64], [65], [66]]. Zhang et al. established the LPS-stimulated myocardial injury rat model, and demonstrated that [67] apigenin substantially decreased the serum levels of myocardial enzymes and inflammatory factors. The underlying mechanism might involve the inhibition of the SphK1/S1P signaling pathway. In the study of Li et al. [36], apigenin alleviated LPS-induced cardiac injuries, tissue damage and cardiomyocyte death, which was implied by the improved indicators of myocardial injury (LDH and CK), cell death (TUNEL staining, DNA fragmentation, and PARP activity), and tissue damage (cTnI and cMLC1), as well as the significantly ameliorated cardiac function (EF and end diastolic LVID). They also noted that these changes might be implicated with antioxidation, anti-inflammation, apoptosis, and autophagy.

### 4.2 Apigenin in sepsis-related liver injuries

As a vital organ responsible for the bioconversion and energy metabolism in the human body, the liver is a prominent site for cytokine production with the most intense inflammatory responses occurring during the development and progression of sepsis. Under sepsis, liver-mediated inflammatory and immune responses are a “double-edged sword” that can lead to inflammatory cascades, immunosuppression and organ damage while clearing bacteria and toxins [68].^.^Zhou et al. established murine liver injury models with D-GalN/LPS and revealed that [69] apigenin could protect the liver, which might be correlated with the up-regulated expression of liver Nrf-2 and PPARγ. In several other studies, apigenin pretreatments lifted serum albumin and total protein levels, liver GSH level, catalase and SOD activities, and decreased the levels of serum ALT, AST, ALP, γ-GT, CRP, total and direct bilirubin, MDA, NOx, PGE2, TNF-α, IL-1β, IL-6, iNOS, COX-2 and mRNA, liver MPO activity, and phosphorylation of NF-κB p65, IκB, and IKK proteins. However, the phosphorylation of p38, ERK, and JNK proteins was not affected. These findings suggested that apigenin pretreatments could ameliorate the LPS-induced acute liver injury [59,70]. The Nrf2/Keap1 signaling pathway is strongly associated with antioxidation. In normal conditions, Nrf2 binds to its inhibitory protein Keap-1 and is retained in the cytoplasm in a non-activated state. Under LPS/D-GalN induction, Nrf2/Keap1 is separated rapidly, with the released Nrf2 translocated to the nucleus and promoting the transcriptional translation of antioxidant substances, such as SOD and HO-1. After apigenin intervention, the expression of Nrf2 and HO-1 is significantly increased, indicating that the relieving effect of apigenin on septic liver injury is potentially correlated with the Nrf2/Keap1 signaling pathway. Besides, compelling evidence was offered demonstrating that such relieving effect was also associated with the NF-κB signaling pathway, apoptotic pathways [71], and PPARγ activation [72].

### 4.3 Apigenin in sepsis-related kidney injuries

Kidneys are susceptible to sepsis, while sepsis is the most common etiology of AKI. SA-AKI occurs in 40%–50 % of septic patients, which results in a higher mortality rate compared with non-SA-AKI. Therefore, it is important to facilitate the early diagnosis and prevention of SA-AKI for better patient prognosis. It was demonstrated that apigenin mitigated the methotrexate silica nanoparticle-induced oxidative stress, hepatotoxicity, and nephrotoxicity, because apigenin evidently decreased the levels of serum BUN and Scr, up-regulated the levels of SOD, GSH and CAT, and improved the pathological changes of the kidney in model mice. The protective role of apigenin in the model mice was concerned with its capacity to elevate antioxidant levels, diminish ROS accumulation, and suppress inflammatory mediator (TNF-α and IL-6) expression. The mechanism underlying such protective effects was potentially associated with the up-regulated activity of FOXO3a, lifted level of IkBα, and attenuated nuclear translocation of NF-κB [73]. Additionally, He et al. reported that [74] miR-140-5p negatively regulated CXCL12, while CXCL12 could facilitate the activation of the NF-κB pathway. Their further observation on the *in-vivo* and *in-vitro* I/R-induced kidney injury model revealed that apigenin up-regulated miR-140-5p and down-regulated CXCL12 to repress the NF-κB pathway, thus alleviating kidney inflammation.

### 4.4 Apigenin in sepsis-related lung injuries

**Fig. 7:**
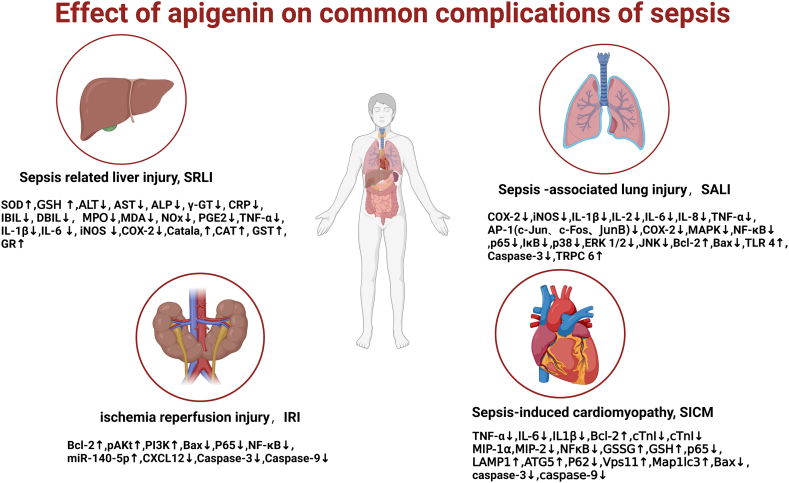
Apigenin has protective effects on heart, liver, lung, kidney and other organs.

ALI is one of the most common complications of sepsis. During the development of ALI, with the pulmonary capillary endothelial injury and increased vascular permeability, a large volume of protein-abundant exudate destroys the pulmonary epithelial cell barrier and accumulates in the alveoli. As a result, effective ventilation is reduced and intractable hypoxemia is triggered, thus leading to pulmonary edema and acute respiratory failure [75]. Apigenin plays an essential part in the treatment of pulmonary inflammatory diseases and exerts the anti-inflammatory effect by repressing the expression of inflammatory mediators and AP-1 factors. Through the administration of LPS-induced model on the A549 cells (human alveolar type II epithelial cells), it was shown that apigenin visibly suppressed the generation of NOx, and the expression of LPS-induced iNOS, COX-2, proinflammatory cytokines (Il-1β, IL-2, IL-6, IL-8, and TNF-α), and AP-1 proteins (c-Jun, c-Fos, and JunB) [76]. In the study of Li et al., the *in-vitro* RAW264.7 macrophages administered with LPS exhibited affected and changed pulmonary pathology, MPO activity, total PMN level, cytokine level in BALF and AOE activity. Apigenin played an anti-inflammatory role through the MAPK and IκB pathways, thereby markedly inhibiting lung tissue inflammation [77]. Furthermore, Kunping Li et al. reported that [62] apigenin attenuated LPS-stimulated ALI by modulating the TLR4/TRPC6 pathway, reducing the release of pro-inflammatory cytokines, and regulating the expression of apoptosis-related factors. （Fig. 7）(Table 1)

**Table 1:** Treatment of apigenin in sepsis.

Possible mechanisms	Ingredient	Model	Dose	Target	References
anti-inflammatory	Apigenin	LPS culture *in vitro*	10 μM–50μM	ICAM-1↓, VCAM-1↓， NO↓, COX-2↓	[18]
anti-inflammatory	Apigenin	CLP Mouse experiment	40 mg/kg	TNF-α↓, TGF-β↓, IL-1β↓, IL-6↓, IL-10↑, polymorphonuclear leukocytes (PNLs)↓, edema↓, congestion↓, hemorrhagia↓Bax↓， Caspase-3↓Lipid peroxidase (LPO)↓，superoxide dismutase (SOD)↑， catalase (CAT) activities↑， glutathione (GSH) levels ↑	[19]
anti-inflammatory	apigenin	CLP Mouse experiment	60 mg/kg	TNF-α↓,IL-1-β↓,IL-6↓，IL-10↑,NF-κB (p65)↓,LPO↓，SOD↓,GSH↓	[20]
anti-inflammatory	apigenin	LPS culture *in vitro*	50 mg/kg	IL-6↓，TNF-α↓	[21]
anti-inflammatory	Apigenin	LPS culture *in vitro*	30 mm	TNF-α↓，IL-10↑	[22]
anti-inflammatory	Apigenin-7-Glucuronide	LPS culture *in vitro*	1000 μg/mL	NO↓，TNF-α↓	[23]
anti-inflammatory	Apigenin	LPS culture *in vitro*	10 μM	P65↓，IKK↓，NF-κ B↓，TNF-α↓，IL-1-β↓，IL-8↓，	[24]
anti-inflammatory	Apigenin	LPS culture *in vitro*	40 μM	NF-κB↓，GSK 3 β/Nrf 2↑，HO-1↑，TNF-α↓，IL-1β↓，IL-6↓	[25]
anti-inflammatory	Apigenin	LPS culture *in vitro*	20 μM	Akt↓，mTOR↓，JNK↓，p38-MAPK↓，NF-κB↓，IL-1β↓，IL-6↓，COX-2↓	[28]
anti-inflammatory	Apigenin	LPS culture *in vitro*	20 μM	AGEs↓，ICAM 1，↓ROS↓，NF-κB↓，Nrf 2↑，MGO↓，MCP-1↓，IL6↓ERK/NF-κB↓	[31]
antioxidation	Apigenin	LPS Mouse experiment	50 mg/kg	LDH↓, CK↓，TNF-α↓，IL-1 β↓MIP-1 α↓， MIP-2↓， LAMP1↑，ATG5↑， p65↓，p62↓ ，Vps11 ↑，Map1lc3↑，cTnI↑，cMLC1↓， GSSG ↑,GSH↓，NF-κB↓，TFEB↑	[36]
Antioxidation apoptosis	Apigenin	LPS culture *in vitro*	0.1 μM	Caspase-3↓，ROS↓，NF-κ B↓，p-ERK↓，p-JNK↓，p-p38↑	[37]
apoptosis	Apigenin	LPS Mouse experiment	50 mg/kg	NF-κ B↓，p65↓，MIP-2↓	[60]
apoptosis	Apigenin C-Glycosides	LPS Mouse experiment	40mgKg	TNF-α↓，IL-6↓，IL-1β↓，MAPK↓，p38↓，ERK 1/2↓，JNK↓，Bcl-2↑，Bax↓，caspase-3↓，TLR 4↑，TRPC 6↑	[62]
Protect the heart	Apigenin	LPS Mouse experiment	100 mg/kg	TNF-α↓、IL-6↓、IL-1b↓caspase-3↓， caspase-9↓，Bax↓，Bcl-2↑	[67]
protect liver	Apigenin	LPS Mouse experiment	100 mg/kg	SOD↑，GSH ↑，ALT↓, AST↓, ALP↓, γ-GT↓, CRP↓, total and direct bilirubin levels, ↓liver MPO↓ activity, MDA↓, NOx↓, PGE2↓, TNF-α↓, IL-1β↓，IL-6 ↓, iNOS ↓ COX-2↓	[70]
protect liver	Apigenin	LPS Mouse experiment	100 mg/kg	iNOS↓, MDA↓catalase (CAT)↑, SOD↑, TAOC↑, Nrf2↑，HO-1↑ALT↓, AST↓，NF-κB↓，IKK↓，IκBα↓，NF-κB/p65↓，Caspase↓	[71]
protect liver	Apigenin	LPS Mouse experiment	200 mg/kg	Nrf-2↑,PPARg↑,NF-kB↓,p65↓,TNF-α↓, ALT↓, AST↓, SOD↑, CAT↑, GST↑, GR↑	[72]
Protect the lungs	Apigenin	LPS culture *in vitro*	40 μM	COX-2↓,iNOS↓,IL-1β↓,IL-2↓,IL-6↓,IL-8↓, TNF-α↓,AP-1（c-Jun、c-Fos、JunB）↓	[76]
Protect the lungs	Apigenin-7-Glycoside	LPS Mouse experiment	10 mg/kg	MAPK↓, NF-κB（IκB）↓,TNF-α↓,IL-6↓,IL-1β↓, COX2↓,iNOS↓,p38MAPK ↓,NF-κB p65↓,IκB↓	[77]

## 5 Limitations and prospects of apigenin in septic treatment

Despite the various functions of apigenin including anti-inflammation, anti-cancer, anti-oxidative stress, pro-autophagy, anti-apoptosis, anti-bacteria, and anti-virus, there are still some problems regarding the application of apigenin. Firstly, the existing studies on the role of apigenin in septic treatment were all conducted based on animal or cell experiments, whereas its therapeutic role in clinical patients and the underlying mechanism remains ambiguous. The information such as its preventive functions, bioavailability, and bioactivity in human bodies is hard to acquire, and its pharmacological effects, adverse reactions, and safety in humans are all unknown [78]. Secondly, apigenin is present in food in the form of glycosides with low water solubility and poor oral bioavailability. After ingestion, most apigenin is either excreted without being absorbed, or metabolized rapidly after absorption without exhibiting the *in-vivo* activity [79]. In this context, novel carriers are required to improve the oral bioavailability of apigenin. Some carriers have already been adopted. For example, nano-sized drug carriers have been found to be able to effectively enhance the oral bioavailability of apigenin [80,81], and the self-microemulsifying drug delivery system has also been reported to improve the solubility and dissolution of apigenin [82]. The nanoemulsifying technique doubles the peak apigenin concentration in the blood, enhances the absorption of apigenin, and prolongs the retention time of apigenin in the body [83]. In addition, the specific drug target of apigenin in sepsis is yet to be determined, thus it is crucial to identify and validate the target. Following the elucidation of target genes, it is imperative to combine targeted drugs with traditional sepsis drugs to optimise treatment efficacy. Finally, apigenin can be utilized either in the form of food additives or as drugs. However, toxicological and relevant clinical investigations are still needed to confirm the safety and efficacy of apigenin in the treatment of sepsis-related complications, so as to adopt apigenin as a drug. Therefore, more and more in-depth research work is needed to truly apply apigenin in the treatment of sepsis.

## 6 Conclusion

Sepsis, known as a typical disease in modern times, is one of the leading causes of disabilities and deaths. The pathogenesis of sepsis is a complex process and early diagnosis plays a crucial role in meeting the needs patients. The management of sepsis has received much attention in recent years. Numerous studies that require considerable time and money have been carried out in order to explore the mechanism of sepsis and to figure out an optimal treatment against the disease. Nevertheless, the most radical and effective treatment has not been identified yet. Apigenin, as a natural flavonoid, is widely found in people's daily diet, as it can be regulated by daily diet to play a role in disease prevention and treatment. In addition, Apigenin has been widely applied due to its anti-inflammatory, anti-cancer, antioxidant, and anti-viral effects. Currently, accumulating evidence has been provided by epidemiological and case-control research to demonstrate that a higher intake of phyto-flavonoids is associated with a lower risk of contracting chronic diseases. Compared with other flavonoids, apigenin is non-toxic and non-mutagenic, which has attracted extensive attention from researchers focusing on sepsis because of its anti-inflammatory, antioxidant, pro-autophagic, anti-apoptotic, immune cell-protective, and organ perfusion injury-alleviating effects. The above studies have shown that apigenin inhibits the development of sepsis *in vitro* and *in vivo* through mechanisms such as anti-inflammatory, antioxidant, macrophage polarization, autophagy and endothelial cell protection. In addition, studies have shown that apigenin has an important role in sepsis-related complications. The plethora of *in vitro* and *in vivo* protective effects of apigenin against sepsis offers great potential for its application in sepsis therapy. In this context, the current investigation presents an opportunity for the further exploration of the effects of apigenin in clinical septic management. Since most of the studies on the biological activity of apigenin is mostly based on basic research and relatively few clinical trials, the mechanism of action for apigenin remains unclear. We need to further identify the potential targets of apigenin, as well as its pharmacological effects and mechanism of action in the treatment of sepsis. This will accelerate the research and development of apigenin and enable its earlier clinical application.

## Data availability statement

Data availability is not applicable to this article as no new data were created or analyzed in this study.

## Funding

None.

## Availability of data and materials

The data used to support the findings of this study are available from the corresponding author upon request.

## Additional information

No additional information is available for this paper.

## CRediT authorship contribution statement

**Lin Zhu:** Writing – original draft. **Hairong Zhang:** Writing – original draft, Resources. **Xiaoyu Zhang:** Writing – original draft, Visualization, Investigation. **Lei Xia:** Writing – review & editing, Resources. **JiaJia Zhang:** Writing – review & editing, Funding acquisition.

## Declaration of competing interest

The authors declare that they have no known competing financial interests or personal relationships that could have appeared to influence the work reported in this paper.
